# Non-hygroscopic ionogel-based humidity-insensitive iontronic sensor arrays for intra-articular pressure sensing

**DOI:** 10.1093/nsr/nwae351

**Published:** 2024-10-03

**Authors:** Junli Shi, Sai Xie, Zhiguang Liu, Minkun Cai, Chuan Fei Guo

**Affiliations:** Department of Materials Science and Engineering, Southern University of Science and Technology, Shenzhen 518055, China; Department of Materials Science and Engineering, Southern University of Science and Technology, Shenzhen 518055, China; Department of Materials Science and Engineering, Southern University of Science and Technology, Shenzhen 518055, China; Department of Materials Science and Engineering, Southern University of Science and Technology, Shenzhen 518055, China; Department of Materials Science and Engineering, Southern University of Science and Technology, Shenzhen 518055, China; Guangdong Provincial Key Laboratory of Functional Oxide Materials and Devices, Southern University of Science and Technology, Shenzhen 518055, China

**Keywords:** iontronic pressure sensor, phase separation, humidity-insensitive ionogel, biocompatibility, intra-articular pressure sensing

## Abstract

Implanted pressure sensors can provide pressure information to assess localized health conditions of specific tissues or organs, such as the intra-articular pressure within knee joints. However, the prerequisites for implanted sensors pose greater challenges than those for wearables or for robots: aside from biocompatibility and tissue-like softness, they must also exhibit humidity insensitivity and high-pressure resolution across a broad pressure spectrum. Iontronic sensors can provide superior sensing properties, but they undergo property degradation in wet environments due to the hygroscopic nature of their active component: ionogels. Herein, we introduce a humidity-insensitive iontronic sensor array based on a hydrophobic and tough ionogel polymerized in a hydrophobicity transition yielding two hydrophobic phases: a soft liquid-rich phase that enhances ionic conductivity and ductility, and a stiff polymer-rich phase that contributes to superior toughness. We demonstrate the *in vivo* implantation of these sensor arrays to monitor real-time intra-articular pressure distribution in a sheep model, while assessing knee flexion with an angular resolution of 0.1° and a pressure resolution of 0.1%. We anticipate that this sensor array will find applications in various orthopedic surgeries and implantable medical devices.

## Teaser

A humidity-insensitive iontronic sensor array using a nonhygroscopic phase-separated ionogel and bonded interfaces is used for high precision intra-articular pressure sensing.

## INTRODUCTION

A large number of people are suffering from knee-joint problems, and severe knee conditions can benefit from knee-joint surgery [1–3]. Knee-joint surgery is a medical procedure that is aimed at recovering the function of a pathological knee joint and alleviating its pain through surgical intervention, including arthroscopic surgeries, anterior cruciate ligament reconstruction, meniscus repair or removal surgeries and knee-replacement surgeries [4,5]. The World Health Organization estimates that millions of people worldwide undergo knee-replacement surgery each year [6]. However, ∼10%–20% of surgical patients who have knee-joint surgery are dissatisfied and the dissatisfactory rate is increasing [7,8], mostly due to leg imbalance [9] with an angle deviation of >1° and a joint gap of <1 mm [10,11]. Real-time monitoring of intra-articular pressure is a promising technology to correct the assembly deviations that occur during knee-replacement surgeries for precise alignment of the joints [12,13].

The knee joint is curved surface that is bathed in a synovial fluid and often imposed with high pressure [14,15]. An ideal format of sensors for intra-articular pressure sensing should be a soft and thin layer that can be implanted in the narrow and curved gap of the joints (Fig. S1), without being affected by the fluid. Flexible iontronic pressure sensors are a class of emerging devices that exhibit high sensitivity over a wide range [16–21]. Such sensors are often a trilayer with two flexible electrodes sandwiched by a soft ionogel, forming a nanoscale electric double layer (EDL) [22,23] at the electrode–ionogel interface (Fig. S2). However, existing iontronic sensors are humidity-sensitive and cannot be used in wet environments because ionogels are often hygroscopic, absorbing water in the air or in humid environments and leading to a dramatic signal drift of iontronic sensors [24]. On the one hand, hydrated ionogels undergo a substantial change in electrical properties that leads to the unstable sensing performance of sensors. On the other hand, the mechanical properties of ionogels, such as toughness and modulus, decay under humid conditions. Such a degradation in toughness and modulus causes poor mechanical stability and a narrow working range of the sensors. The hygroscopicity of ionogels, therefore, prevents the usage of iontronic pressure sensors as implants for stable pressure measurement during knee-replacement surgeries and other in-body applications.

Herein, we report a humidity-insensitive, wide-range flexible pressure sensor array for stable intra-articular pressure measurement based on a bicontinuous, non-hygroscopic and tough ionogel. The ionogel is synthesized in a phase-separation polymerization process that yields two hydrophobic phases: a soft liquid-rich phase and a hard polymer-rich phase. The former provides high ionic conductivity and high ductility, and the latter provides a high Young's modulus (107 MPa) for a wide and linear sensing range (0–2 MPa), and both contribute to the humidity insensitivity. The ionogel also exhibits a high activation energy so that the sensor exhibits high chemical stability without signal degradation over 43 years, based on an accelerated aging test. A sensor array with 26 sensors was further implanted into the knee joints of an *in vivo* sheep model and our sensory system provided real-time and high-precision detection of intra-articular pressure and joint imbalance. This work provides a platform for stable and accurate intra-articular pressure measurement and also for other biomedical applications in wet and high-pressure environments.

## RESULTS AND DISCUSSION

### Flexible pressure sensors for intra-articular pressure measurement

**Figure 1.**
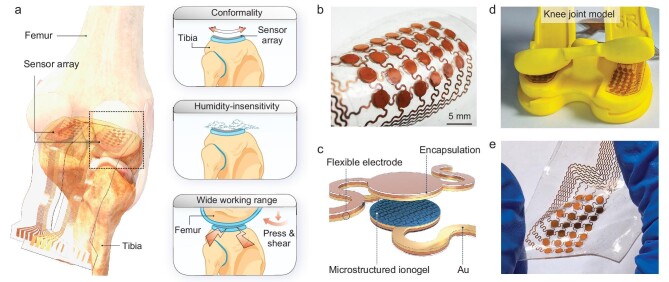
Challenges of intra-articular pressure measurement and flexible iontronic pressure sensor array used in the measurement. (a) Conditions required for intra-articular pressure measurement: sensing on a curved surface, under highly humid conditions and under high pressure and shear. (b) Photograph of the flexible iontronic pressure-sensing array. (c) Schematic showing the structure of the sensor array. (d) The flexible iontronic pressure sensor array can be laminated on curved surfaces of a knee-joint model. (e) Photograph of the flexible iontronic pressure sensor array when stretched to 30%.

The knee joint is a curved structure that is filled with a liquid-like synovial capsule and often subjected to high pressures. Intra-articular pressure measurements are thus challenging because the implanted sensors in a knee joint should be insusceptible to the curvature, insensitive to humidity and able to detect pressure over a wide range (Fig. 1a). Here, we use a soft iontronic sensor array with a hydrophobic and tough ionogel for humid-insensitive and wide-range intra-articular pressure sensing and a stretchable bridge-stiff-island structure to eliminate the interference of the joint curvature. Each sensing unit in the sensor array consists of five layers (Fig. 1b and c): a top polydimethylsiloxane (PDMS) encapsulation layer, a top polyimide-copper (PI-Cu) electrode, an ionogel layer with one side being microstructured, a bottom PI-Cu electrode, and a bottom PDMS encapsulation layer. The electrodes and the ionogel are cut to be rounded with a diameter of 2 mm, with the electrodes connecting to serpentine wires for large stretchability. Each sensor array has 26 sensing units and can be stably laminated onto a curved surface (Fig. 1d), while the stretchable bridge-stiffer island structure of the sensor array enables insensitivity to in-plane strains or curvatures. The sensors are designed to have the interfaces bonded together to improve the mechanical stability, except for the microstructured interface. Such a seamlessly integrated sensor array can be stretched to 30% without any interlayer delamination or debonding (Fig. 1e). The soft sensor-based technique is substantially different from the traditional method for intra-articular pressure measurement (Fig. S3). Our flexible pressure-assisted monitoring system, in the format of a thin layer that can be filled in the joint, is expected to bypass the need for trial molds, allowing real-time and accurate pressure monitoring for leg balance during surgery.

### Synthesis, mechanical properties and electrical properties of the ionogel

An ionogel is a composite that consists of a polymer matrix and an ionic liquid in the polymer chains. Ionogels are often hydrophilic because ions are highly polar and hygroscopic. Many non-polar elastomers, such as PDMS, cannot mix with ionic liquids because of their mismatch in polarity [25]. Here, we *in situ* synthesize a hydrophobic and tough ionogel using acrylonitrile (AN) and ethyl acrylate (EA) as the monomers and 1-ethyl-3-methylimidazolium bis(trifluoromethylsulfonyl)imide ([EMIM][TFSI]) as the ionic liquid. Before polymerization, the AN and EA monomers are all highly miscible with the ionic liquid to form a clear solution. After polymerization, polyethyl acrylate (PEA) and polyacrylonitrile (PAN) have significantly different miscibility with ionic liquids (Fig. S4).

**Figure 2.**
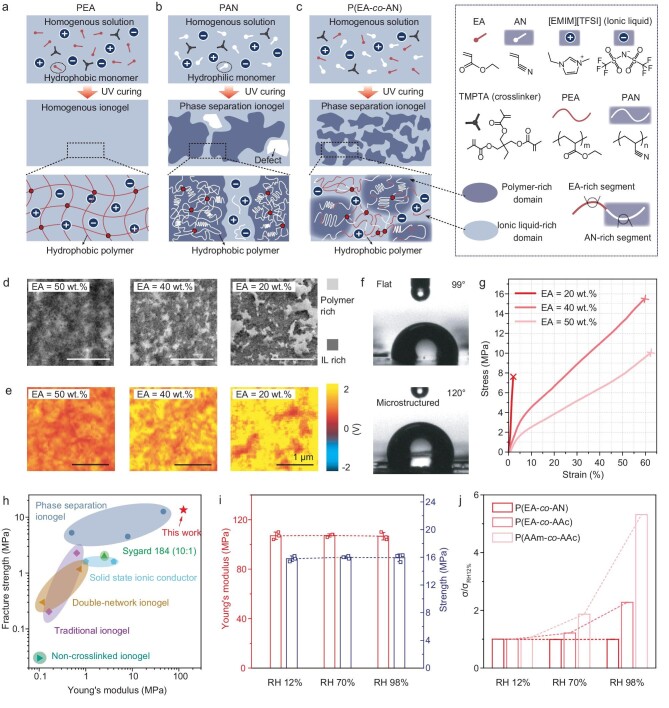
Preparation, mechanical properties and electrical properties of ionogels under different relative humidity levels. (a) Polymerization of single-phased PEA ionogel. (b) Polymerization of the PAN ionogel, which often has internal defects. (c) Phase separation of P(EA-*co*-AN) ionogel. (d) Transmission electron microscopy images of P(EA-*co*-AN) ionogels with EA contents of 50, 40 and 20 wt%. (e) Atomic force microscopy-based infrared spectroscopy (AFM-IR) images of P(EA-*co*-AN) ionogels with EA contents of 50, 40 and 20 wt%. (f) Water-contact angles of P(EA-*co*-AN) ionogel on a flat surface and a microstructured surface, showing the hydrophobic nature of the material. (g) Tensile stress–strain curves of the ionogels with different monomer ratios. (h) Comparison of modulus and tensile strength between our ionogel and the reported results of other ionogels [27–35]. (i) Modulus and tensile strength of the ionogel in different relative humidity levels of RH 12%, RH 70% and RH 98%. (j) Ionic conductivity of our ionogel in reference to that at RH 12% and that of the two control samples, P(EA-*co*-AAc) and P(AAm-*co*-AAc), in different relative humidity levels. IL represents ionic liquid.

PEA is a soft phase that is highly soluble to ionic liquids (Fig. 2a), while polyacrylonitrile (PAN), a highly crystalline polymer (Fig. S5), is almost insoluble to ionic liquids (Fig. 2b). As a result, phase separation of PEA and PAN phases occurs in an *in situ* copolymerization process (Fig. 2c). The structure of the P(EA-*co*-AN) ionogel can be tuned by changing the weight ratio of the monomers. The size of the hard phase increases as the content of the AN increases from 50 to 80 wt% (accordingly, the content of the EA decreases from 50 to 20 wt%), as illustrated in our transmission electron microscopy (TEM) image (Fig. 2d), as well as the atomic force microscopy–infrared spectroscopy (AFM–IR) observation (Fig. 2e) at a wave number of 1570 cm^–1^ (Fig. S6). Furthermore, PEA as an ester and PAN, which contains dense non-polar chains, are both hydrophobic. We show that the P(EA-*co*-AN) ionogel exhibits water-contact angles of 99° on a flat surface and 120° on a microstructured surface (Fig. 2f).

The coexistence of the hard phase and the soft phase achieves a synergistic enhancement of the Young's modulus and tensile strength of the ionogel. The hard PAN phase contributes to the large Young's modulus and strength, while the soft PEA phase contributes to not only ionic conductance, but also high toughness of the material because it enhances the stretchability of the material. Both the strength and the Young's modulus of the ionogel increase as the content of PAN increases from 50 to 60 wt%, with elongation at the break remaining almost unchanged. When the content of the PAN increases to 80 wt%, however, the strong dipole interaction [26] between the cyano groups will lead to the formation of defects in the interior. As a result, the material becomes brittle and its toughness decreases substantially (Fig. 2g). We thus select the composition with 60 wt% PAN for our study because of its high Young's modulus, large stretchability and high toughness of the ionogel (Fig. 2g). Such properties help to achieve a wide sensing range and high robustness of the sensors. Note that our ionogel is even tougher and stiffer than the ‘ultra-tough and stiff ionogel’ of poly(acrylamide-co-acrylic acid) in 1-ethyl-3-methylimidazolium ethyl sulfate [27], single-network cross-linked gels [28,29], double-network gels [30,31], solid-state ionic conductors [32,33] and other phase-separation gels [34,35] (Fig. 2h).

In addition to the excellent mechanical properties, the ionogel also exhibits humidity insensitivity. We compared our ionogel with two other ionogels: a poorly hydrophilic copolymer of PEA and polyacrylic acid (P(EA-*co*-AAc)) with ethyl sulfate-1-methyl-3-ethylimidazole (EMIES) as the ionic liquid, and a highly hydrophilic ionogel prepared using acrylamide (AAm) and AAc as monomers, termed (P(AAm-*co*-AAc)), also with EMIES as the ionic liquid. We show that, under different relative humidity levels of RH 12%, RH 70% and RH 98% for 30 min, both the Young's modulus and the tensile strength of the P(EA-*co*-AN) ionogel remain unchanged (Fig. 2i), while that for the two control ionogels show a substantial decrease in modulus and strength as the relative humidity increases (Figs S7–S9). Specifically, at RH 98%, the fracture energies for P(EA-*co*-AAc) and P(AAm-*co*-AAc) decrease by 90.0% and 94.4% compared with the case at RH 12%. Furthermore, the electrical conductivity of the P(EA-*co*-AN) ionogel is also stable under different humidity conditions. By contrast, both control samples, the P(EA-*co*-AAc) and P(AAm-*co*-AAc) ionogels, exhibit a large increase in ionic conductance (Fig. 2j) and capacitance of a capacitor (Fig. S10) under highly humid conditions due to water absorption.

### Sensing properties of the sensor array

**Figure 3.**
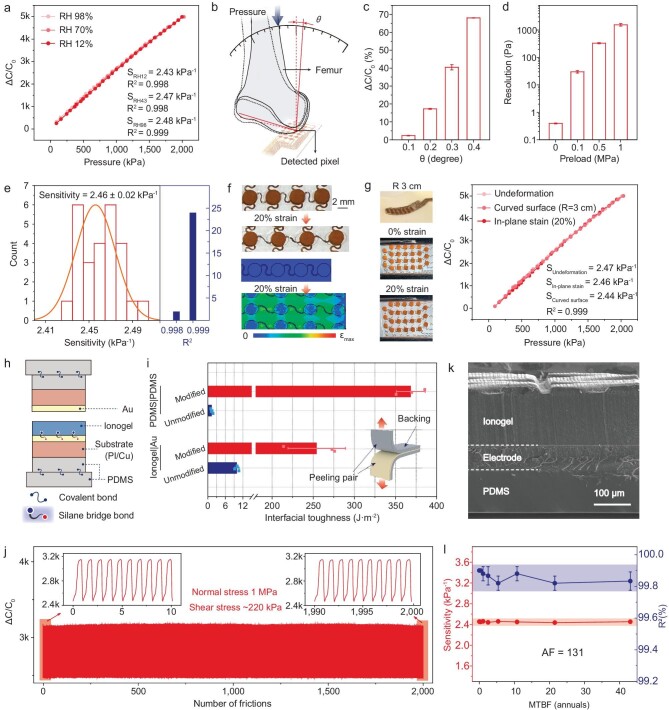
Sensing properties and stability of the flexible iontronic pressure sensor array. (a) Response curves of a single sensing unit under different relative humidity levels of RH 12%, 70% and 98%. (b) Schematic diagram of the set-up for the test of angular resolution. (c) Response of the sensor to angular changes of 0.1°, 0.2°, 0.3° and 0.4°. An angular resolution of ≥0.1° is determined. (d) Pressure resolutions of the sensor under different preloads of 0, 0.1, 0.5 and 1.0 MPa. (e) Statistic distribution of sensitivity and linearity values of 26 pixels in a sensor array. (f) Photographs and simulation results of a sensor array stretched from 0% to 20%. (g) Capacitance–pressure responses of a single sensing unit under no in-plane strain, subjected to in-plane strain of 20% and laminated on a curved surface, showing that the response is insensitive to in-plane strain or curvature. (h) Schematic of the layered structure of the sensor. (i) Interfacial toughness of the covalently bonded interfaces in panel (h). Without interfacial bonding, the interfacial adhesion is much weaker. (j) Response of a sensing unit under repeated rubbing of 2000 cycles. The applied pressure is 1 MPa and the shear stress is 220 kPa. (k) Cross-sectional view scanning electron microscopy image of the sensor after rubbing test. No delamination between the interfaces is observed. (l) Sensitivity and linearity over mean time between failure (MTBF) of a sensing unit measured in an accelerated aging test. The acceleration factor (AF) is 131. Both sensitivity and linearity remained almost unchanged over the test (43 annuals).

We used the hydrophobic and tough ionogel as the active layer in a sensor array. Sensitivity, sensing range and linearity are key parameters of flexible pressure sensors. Sensitivity *S* is defined as *S*=*δ*(*ΔC/C*_0_)/*δP*, where *C* represents the instantaneous capacitance, *C*_0_ represents the initial capacitance before loading and *P* represents the applied pressure. We tested the sensitivity of a selected sensing unit in an array under three relative humidity levels (RH 12%, RH 70% and RH 98%). The results show that the sensing units exhibit constant and close sensitivity values of 2.43, 2.47, and 2.48 kPa^–1^ (Fig. 3a), all with high linearity (*R*^2^ > 0.998) in a wide range of 0–2.0 MPa. The wide range and linear response is related to both the structure design and the large Young's modulus of the ionogel. The microstructure—a pillar-like structure with synergetic gradients in the width and height directions—is developed by using a machine-learning model for a linear response [36]. The linear range is further widened by using the ionogel with a large Young's modulus, although there is often a trade-off between linear range and sensitivity (Fig. S11).

Both angular resolution and pressure resolution are important for intra-articular pressure-sensing applications. Angular resolution is defined as the minimal rotational change of angle that the array can resolve. We built a set-up to detect the angular resolution by imposing a force on an artificial femur to press a sensor array and change its inter-axis angle with an increment of 0.1° at an initial angle of 90° (Fig. 3b). We show that the rotation can be detected from a selected pixel in the array, indicating an angular resolution of 0.1° (Fig. 3c). Besides, the limit of detection of the sensor array is determined to be 0.38 Pa and the pressure resolution at preloads of 100, 500 and 1000 kPa are determined to be 32 Pa, 422 Pa, and 1.55 kPa, respectively (Fig. 3d). Such high angular resolution and pressure resolution enable the precise measurement of intro-articular pressure of our sensor array.

The sensing properties of the sensing units are highly uniform. We tested all 26 pixels in an array and the results show a small sensitivity difference of only 0.8%, with all sensing units exhibiting high linearity (*R*^2^ > 0.998) (Fig. 3e). The deviation is even smaller than that of commercial silicon-based sensors [37,38]. The high uniformity stems from the contact mode of iontronic sensing—the signal magnitude is determined by the interfacial contact area rather than the thickness of the ionogel [39]. A small difference in thickness of the ionogel will not affect the response of the iontronic sensor. By contrast, the signal magnitude of conventional capacitive sensors highly relies on the thickness control of the dielectric layer, for which the deviation is difficult to control.

### Conformability and strain insensitivity of the flexible pressure sensor array

Serpentine interconnects have been proven to be effective to achieve large stretchability and conformability of electronic devices [40–42]. Here, the sensor array was designed to have a stretchable bridge-stiff-island structure [43]. The islands are sensing units that are made of a rigid PI-Cu/ionogel/PI-Cu trilayer, with all materials being bendable but the trilayer being not stretchable (Fig. 3f). The bridges are flexible and stretchable serpentine interconnects, encapsulated by PDMS layers. Upon stretching, only the serpentines and the PDMS encapsulation layers are elongated, while negligible deformation of the sensing units occurs. Such a structure helps to eliminate the response of the sensing units to in-plane strains. We show that the capacitance–pressure response of a sensing unit does not change when it is stretched from 0% to 20%, and no signal is detected when the sensor array is subjected to in-plane strains or covered on a curved surface, including the curved surface of a joint (Fig. 3g).

### Mechanical and chemical stability of the sensor array

The mechanical stability of the sensor array should be considered, as the knee joints are often subjected to both high shear stress and high pressure. We use interfacial bonding to improve the mechanical stability of the sensor array (Fig. 3h). Specifically, a monolayer of 3-mercaptopropyl-trimethoxysilane (MPTMS) containing a mercapto group and a monolayer of 3-(trime-thoxysilyl)propyl methacrylate (TMSPMA) containing an unsaturated double bond were used to modify the surface of the Au-coated PI-Cu electrode (Fig. S12). The Au layer and the thiol groups of the MPTMS monolayer form a strong Au-S interaction [44,45] and the two monolayers are bonded via a condensation reaction. The unsaturated double bonds of TMSPMA are exposed, which build a strong bond with the C=C bond in the EA and AN monomers during the photo-polymerization process. Besides the adhesion between the ionogel and the Cu electrode, the two PDMS encapsulation layers are plasma-treated and bonded via the formation of Si–O–Si covalent bonds for sealing [46,47]. Such modification greatly improves the mechanical stability of the interfaces: the interfacial toughness between the flat surface of the ionogel and the electrode is as high as 418 J·m^–2^. Without chemical bonding, the interfacial toughness is only 22 J·m^–2^. In addition, the interfacial toughness of the PDMS–PDMS encapsulation layers is 369 J·m^–2^ (Fig. 3i), which is otherwise only ∼1.3 J·m^–2^ without interfacial bonding.

We further explored the fatigue resistance of the sensor array when it is used under high-shear and high-pressure conditions. We randomly select a sensing unit in an array for the cyclic friction test. The results show that the sensor can stably work for 2000 cycles under a combined high pressure of 1.0 MPa and a shear stress of 220 kPa without exhibiting signal drift (Fig. 3j) or interfacial failure (Fig. 3k). By contrast, a control sensor, for which all interlayers are simply stacked without bonding, shows substantial signal drift under combined compression and shear. Delamination between the functional layers is also found (Fig. S13).

The sensors are chemically stable over tens of years under normal working conditions. We performed an accelerated aging test of the sensor array under a humidity–heat aging condition (at RH 98% and 328 K) and also tested the degradation activation energy of the ionogel (*E*_a_) by using a thermogravimetric analyser (Fig. S14). The activation energy was determined to be 0.80 eV from the derivative curves of different heating rates and its mass loss, corresponding to an acceleration factor (AF) of 131 based on the Hallberg–Peck model [48,49]. We tested the responses of four sensors in a sensing array under different aging times and found that the responses did not change over 120 days in the aging condition, corresponding to 43 years under normal working conditions of RH 50% and 298 K (Fig. 3l).

### Biocompatibility of the sensor array

**Figure 4.**
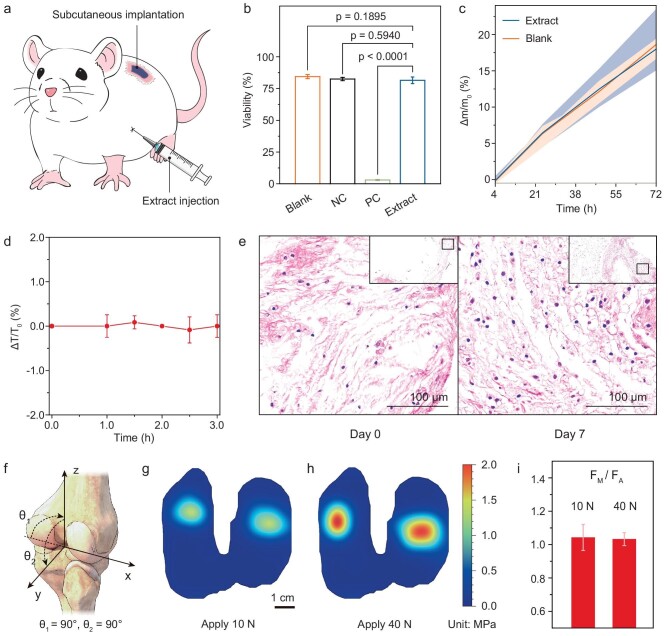
Cytotoxicity, *in vivo* biocompatibility of the sensor array and its validity for pressure measurement in a knee-joint model. (a) Schematic diagram of the *in vitro* and *in vivo* biocompatibility test using extract injection or subcutaneous implantation in a mouse model. (b) *In vitro* cytotoxicity test of the sensor array. Results from blank control, negative control (NC) and positive control (PC) groups are compared. The survival rates are all >87.6%, indicating that the sensor array has no significant toxicity. (c) Mass change of a mouse injected with a sensor array extract in an acute systemic toxicity test with its error range. The result is close to that of the control model without the extract injection. Δ*m* is the change in mass and *m*_0_ is the original mass before the test. (d) Temperature change of a mouse for a pyrogen test, where Δ*T* is the change in temperature and *T*_0_ is the original temperature before the test. (e) *In vivo* biocompatibility test of the arrays by histological observation of tissue slices after implanting for 7 d. (f) Schematic diagram of an *in vitro* bone model for intra-articular pressure test. (g) The pressure mapping of the tibia plane with 10 N of vertical stress and (h) 40 N of vertical stress. (i) Ratios of measured force to applied force under loads of 10 and 40 N. Both values are close to 1.0.

The biocompatibility of the sensor array has been studied to confirm its potential applications in joints. We evaluated the biocompatibility by conducting *in vitro* cytotoxicity, acute toxicity and pyrogen tests, as well as an *in vivo* inflammation test through histological observation. The *in vitro* cytotoxicity test was conducted by extract injection or by subcutaneous implantation (Fig. 4a) and a pressure sensor array was used for test sample extraction. First, L-929 cells were digested by using trypsin with a cell suspension of 1 × 10^5^ cells per milliliter and then cultured in an incubator at minimum essential medium (MEM) with 10% fetal bovine serum. After the cells had grown into a monolayer, the original culture medium was aspirated and 100 mL of test sample extracts (concentrations of 100%, 75%, 50% and 25%), blank control solution, positive control solution and negative control solution were further cultured at 37°C in 5% CO_2_ for 24 h. After culturing, the cell morphology was observed by using fluorescence microscopy and its absorbance at 570 nm was measured (reference wavelength: 650 nm) on a microplate reader to observe the cell survival rate. The results show that the survival rates are all >87.6%, indicating that the sensor array has no significant toxicity to L-929 cells (Fig. 4b).

Acute toxicity was also tested by injecting the test sample extract and negative control solution. The selected extraction solvent was 0.9 wt% sodium chloride injection, the extraction ratio was 3 cm^2^ per milliliter and the injection dose was 50 mL kg^–1^. No significant difference in weight between the experimental animal and the control animal was observed, indicating that the polar extract of the test sample does not cause acute toxicity (Fig. 4c). A similar operation of injecting extract was used to perform the pyrogen test and there was no temperature difference between the experimental and the control animals (Fig. 4d). The results are in accordance with the pyrogen test regulations.

We further used hematoxylin–eosin staining to evaluate inflammation through the subcutaneous implantation of a sensor array in a mouse model by a blinded pathologist. Histological assessment showed that both the control sample and the experimental sample had mild inflammatory cell infiltration after 1 week. The degree of inflammation at the implantation site with the control sample and with the sensor array received an average score of 0.5 and 1, respectively—all falling in the ‘very mild’ inflammation range (Fig. 4e). The result is satisfactory for a short period of implantation in joints.

### *In vitro* pressure measurement of the sensor array in a knee model

We used the sensor array for pressure measurement in an *in vitro* prosthetic knee model (Fig. 4f). A pressure sensor array was placed in the joint of a prosthetic knee model and the signal of each channel was recorded when a force was applied (Fig. 4g and h). Under applied normal forces of 10 and 40 N, the measured force (*F*_M_, by summing signals from all channels) was compared with the applied force (*F*_a_). *F*_m_ can be calculated by using Equation (1):


(1) \begin{eqnarray*}
{{F}_m} = \mathop \sum \limits_{i = 1}^{26} {{P}_i} \cdot A,
\end{eqnarray*}

where *P*_i_ is the pressure value of each pixel that can be measured by sensor number *i* (as shown in Fig. 3e) and *A* is the area of a single pixel. The pressure is applied only to the sensing areas rather than the gaps between the sensors (Fig. S15). *F*_m_ was found to match well with *F*_a_ in both cases (Fig. 4i). The results show that the sensor array can accurately measure the load applied to the joint.

### Real-time and *in vivo* intra-articular pressure recording

**Figure 5.**
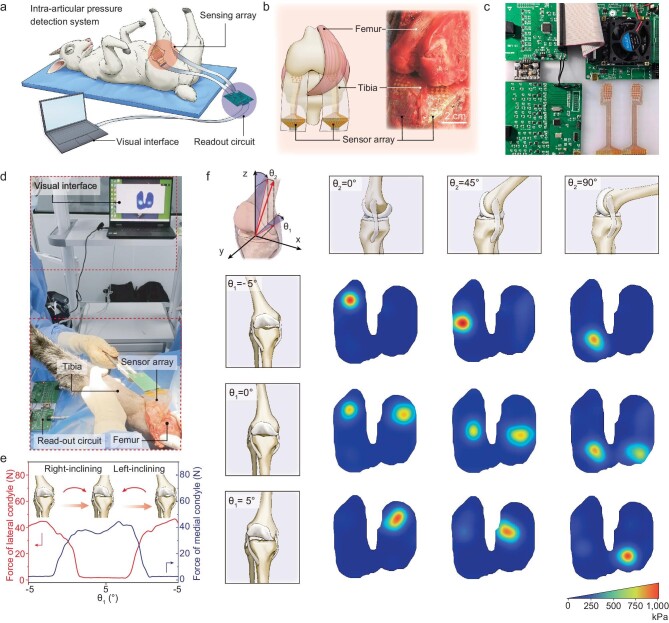
*In vivo* intra-articular pressure detection in a sheep model using flexible iontronic pressure sensor arrays. (a) Schematic diagram of intra-articular pressure detection using a sensory system in a sheep model. The sensory system includes two sensor arrays—a circuit and a computer with a real-time visual interface showing pressure distribution. (b) Schematic and photograph for the implantation of two sensory arrays between the femur and the tibia of a knee joint. (c) Photograph of the readout circuit. (d) Photograph of the *in vivo* intra-pressure measurement of a knee joint in a sheep model. (e) Detected force of the lateral and medial condyles when rotating the tibia from –5° to +5°. (f) Intra-articular pressure mapping of nine states when the femur changes from the extension position (*θ*_2_ = 0°) to the middle position (*θ*_2_ = 45°) and to the flexion position (*θ*_2_ = 90°) and angel *θ*_1_ changes from –5° to 0, and to +5°.

We further constructed an intra-articular pressure measurement system (Fig. 5a) for real-time and *in vivo* pressure recording, as our sensor array presents high compatibility and accuracy. The intra-articular pressure measurement system contains two sensor arrays for the lateral condyle and the medial condyle (Fig. 5b), respectively, together with their accompanying readout circuit (Fig. 5c). The readout circuit uses a method called ‘frequency division multiplexing’ for a signal readout—each sensing unit is read using a separate and encoded frequency to avoid interpixel interference and crosstalk, given that the response of iontronic sensors is frequency-dependent [50]. The orthogonal frequency is propagated to the decoder by using a capacitor–voltage converter and the real-time, crosstalk-free signal acquisition of the sensor array is realized by using a field programmable gate array (Fig. S16).

The sensor arrays were sutured on the tibia surface (Fig. S17) of a sheep model using bone screws by a surgeon for pressure recording (Fig. 5d). The temperature during implantation was consistent with that of the animal model, at ∼38.5°C. Furthermore, the sensor was fully submerged in a synovial fluid, making humidity insensitivity essential to ensure its reliability in such an environment. We slowly rotated the femur from the lateral to the medial condyle side in an angular range of –5° to +5° to record the real-time intra-articular pressure during the rotation and we show that the signals from the two sensor arrays all change with the rotational angle, with the signal amplitudes of the two arrays being supplementary (Fig. 5e). We further define a coordinate system for the knee joint, with the *x*-axis situated along the two articular fossa, the *y*-axis situated along the articular surface and perpendicular to the *x*-axis, and the *z*-axis being perpendicular to both the *x*-axis and *y*-axis. The interaxial angles of the tibial orientation are defined as *θ*_1_ in the *x*–*y* plane and *θ*_2_ in the *x*–*z* plane (Fig. 5f). We tested the real-time pressure distribution of nine combined states with *θ*_1_ of –5°, 0 and +5°, and *θ*_2_ of 0, 45° and 90° by rotating the femur (Fig. S18). Our system can visually display the real-time pressure distribution of the nine states and the results show that the pressure is not uniformly distributed on the joint surfaces, but rather is concentrated. Furthermore, the tilt angle significantly affects the balance of the lateral and medial condyles, and thus our system can be potentially used to provide visual information for imbalance correction. We expect that our real-time pressure measurement system can be used for unbalanced pressure correction in not only knee joins, but also many other articular joints.

## MATERIALS AND METHODS

Detailed materials and methods are available in the Supplementary data.

## Supplementary Material

nwae351_Supplemental_File
