# Supplementary material for: Non-hygroscopic ionogel-based humidity-insensitive iontronic sensor arrays for intra-articular pressure sensing
Source: Natl Sci Rev. 2024 Oct 3;11(11):nwae351. doi: 10.1093/nsr/nwae351 (PMC11536762; doi:10.1093/nsr/nwae351)
Supplement: nwae351_Supplemental_File [file nwae351_supplemental_file.pdf]

Supporting Information for

Non-hygroscopic ionogel-based humidity-insensitive iontronic sensor arrays for intra-articular pressure sensing

Junli Shi[1,†], Sai Xie[1,†], Zhiguang Liu[1], Minkun Cai[1], Chuan Fei Guo[1,2,*]

[1]Department of Materials Science and Engineering, Southern University of Science and Technology, Shenzhen 518055, China.

[2]Guangdong Provincial Key Laboratory of Functional Oxide Materials and Devices, Southern University of Science and Technology, Shenzhen 518055, Guangdong, China.

***Corresponding author.** E-mail: guocf@sustech.edu.cn

[†]Equally contributed to this work.

Materials and Methods

Synthesis of the P(EA-co-AN) ionogel

The P(EA-co-AN) ionogel was synthesized in a copolymerization process. First, 0.013 g (0.5 wt.% compared to monomer) diphenyl(2,4,6-trimethylbenzoyl)phosphine oxide (TPO, 98 vol.% in purity, TCI) was added into a penicillin bottle as the initiator. Next, ethyl acrylate (99 vol.% in purity, Aladdin) of 0.80 g and acrylonitrile (99 vol.% in purity, Aladdin) of 1.2 g was added into the bottle, followed by adding 2.0 g (100 wt% compared to monomer) 1-ethyl-3-methylimidazolium bis (trifluoromethylsulfonyl) imide ([EMIM][TFSI], 97% in purity, Aladdin). After that, 1.5 g (30 wt.% compared to monomer) trimethylolpropane triacrylate (TMPTA, 98 vol.% in purity, Aladdin) which served as a crosslinker was added into the mixture. The mixture was oscillated until the solution became clear, and the solution was instilled into the gap (100 nm) between two glass plates wrapped with a polyethylene-based release film on their surfaces, followed by ultraviolet curing in an ultraviolet crosslinker (CL-1000L, AnalytikJena) at a wavelength of 254 nm at 8 W for 30 min. The cured P(EA-co-AN) ionogel was stewed in an oven at 40 ℃ for 12 h for the evaporation of unreacted monomers.

Synthesis of control hydrophilic ionogels P(EA-co-AAc) EMIES and P(AAm-co-AAc) EMIES.

For the synthesis of hydrophilic control sample P(EA-co-AAc) EMIES, 0.01g TPO was first added into a penicillin bottle as the initiator, and 0.4 g EA, 1.6 g acrylic acid (AAc, 99 vol.% in purity, Aladdin) were added in the bottle. Afterwards, 2.0 g (100

wt.% compared to monomer) of 1-ethyl-3-methylimidazolium ethyl sulfate([EMIES], 99 vol.% in purity, RhawnSeal) as the ionic liquid, and 0.4 g of TMPTA as the crosslinker were added into the bottle to form a precursor solution of P(EA-co-AAc) EMIES ionogel. For the synthesis of P(AAm-co-AAc) EMIES, a similar procedure was used, but the EA monomer was replaced with acrylamide (AAm, Aladdin) of 1.6 g, and mass of AAc was 0.4 g. Next, 3.0 g of EMIES was added into the monomers, followed by adding 0.25 g TMPTA as the crosslinker. The curing processes for the two ionogels were the same as that of the P(EA-co-AN) ionogel.

Surface modification of gold films.

The surface of a glass plate was washed in acetone (99.5 vol.% in purity, Shang Lingfeng Chemical Reagents Co., Ltd.) and ethanol (99.7 vol.% in purity, Shang Lingfeng Chemical Reagents Co., Ltd.) in sequence, followed by drying using a nitrogen flow. Next, a 100-nm-thick gold film was deposited on the surface of the glass plate using electron beam evaporation (TF500, HHV). We prepared two silane solutions for surface modification. The solution, which was prepared for surface modification to graft sulfhydryl groups, contained 45 vol.% deionized water, 45 vol.% ethanol, and 10 vol.% (3-mercaptopropyl) triethoxysilane (MPTMS, 95 vol.% in purity, Aladdin). The other solution, which was used to graft acrylic double bond groups, contained 45 vol.% deionized water, 45 vol.% ethanol, and 10 vol.% 3-(trimethoxysilyl) propyl methacrylate (TMPSMA, 97 vol.% in purity, Aladdin). Before using the two solutions, acetic acid (99.5 vol.% in purity, Shang Lingfeng Chemical Reagents Co., Ltd.) was added dropwise to generate a pH value of 3.0. The

glass plate covered with a Au film was then immersed into the MPTMS solution for 2 min and washed with ethanol and deionized water in sequence for three times, followed by immersing in the TMSPMA solution for 30 min and washing in ethanol and deionized water.

Fabrication of microstructured template.

The microstructured template was printed by the digital light processing technique using a 3D printing machine (nanoArch S130, BMF). The solution of 10 wt.% poly(vinyl alcohol) (PVA, Mw ~145,000, Aladdin) was then casted on the template. After curing at room temperature for 24 h, the PVA layer was demolded, serving as a template. A polydimethylsiloxane (PDMS, Sylgard-184, Dow Corning) template was selected for the preparation of the hydrophilic control sample for comparison. The PDMS precursor, with a base-to-curing agent weight ratio of 10:1, was casted onto the surface of a 3D-printed template and demolded after curing at 80 ℃ for 2 h. This process yielded a transparent template featuring a microstructured surface.

Fabrication of patterned electrode array.

Initially, PDMS (with a base-to-curing agent weight ratio of 10:1) was casted on a PI substrate and subsequently spin-coated at 1000 rpm for 2 min. After the curing of the PDMS, the electrode array was fabricated using a laser cutter (WE-6040, Beyond Laser Co., Ltd.). The pattern was then transferred using water-transfer tape after the residual electrode was peeled off, ensuring that the PDMS side to be exposed. A patterned PET mask, exposed at the pixel positions, was then aligned and attached to the electrode. The sample and the prepared PDMS membrane were exposed to air

plasma at 50 W for 20 s. After removing the mask, the activated components were immediately bonded together at 60 ℃ for 20 min. Finally, the water-transfer tape was removed using water, resulting in a stretchable electrode array.

Fabrication of the sensor array.

The electrode array was placed on a piece of glass plate with the Au layer to be exposed. The surface modification process was repeated to activate the Au film. Subsequently, the ionogel precursor solution was added dropwise to the activated pixels, after which a PVA template was placed on the electrode surface and cured at 365 nm and 8 W for 1 h. Next, the PVA template was peeled off, leaving gels with microstructured surfaces located at the pixels on the bottom electrode array. The top electrode and the bottom electrode containing the ionogels were then exposed to air plasma at 50 W for 30 s. The two electrode arrays were immediately compacted together at 60 ℃ for 20 min, yielding a stretchable pressure sensor array.

Characterization of mechanical properties.

The interfacial toughness was measured as depicted in Figure 3i using a 180° peeling test. The width of the sample was denoted as d. Initially, both sides of the peeling pair were exposed to air plasma at 50 W for 20 s. Two PET membranes (50 μm in thickness), serving as the backing layers, were adhered to the two sides of the peeling pair using a silicone rubber adhesive (Sil-poxy, Smooth-On, Inc.). The peeling pair were loaded by grippers equipped with a force gauge installed on a computer-controlled stage (XLD-20E, Jingkong Mechanical Testing Co., Ltd.). The peeling was carried out at a constant rate of 50 mm min^{-1}. The peeling force, F_C, at

which the peeling force stabilizes, was recorded. The interfacial toughness, Γ, was calculated using the formula $\Gamma=2\ F_C/d$.

For the tensile test, the sample was cut into a dumbbell-shaped specimen and subjected to tension at a constant rate of 50 mm min^{-1}, and the stress-strain curves were recorded. The humidity for storing the ionogel was regulated using saturated salt solution (ASTM E104-20a). The saturated salt solution was placed in a vacuum desiccator, and the ionogel was stored within the desiccator for 12 h before the test.

For the rubbing test, a sensor was fixated on a smart stretching tester (WS150-100), and a piece of abrasive paper (#1,000 with a coefficient of friction of 0.22, AT PRO) was attached to the surface of the opposing pair. A pressure of 1 MPa was imposed to the friction pair, and the stretching tester moved reciprocally with a distance of 2 mm at a speed of 5 mm min^{-1}.

Microstructure characterization.

The phase separation morphology of the ionogel was characterized by transmission electron microscopy (TEM, Hitachi H7650). Atomic force microscopy combined with infrared spectroscopy (AFM-IR) was performed using a NanoIR2 system (Anasys Instruments). Fourier-transform infrared (FTIR) spectroscopy was utilized to detect the functional groups in the polymer, with the spectra recorded using a Bruker Vertex 70v spectrometer. The morphology including cross sectional views of the microstructure of the ionogel, as characterized using cold field emission scanning electron microscopy (Hitachi SU8230). X-ray photoelectron spectroscopy (XPS) spectra were collected using an Escalab Xi+ spectrometer (Thermo Scientific),

equipped with a dual-gun aluminum X-ray radiation source (1486.6 eV) with a 0.85 eV line width.

Sensing properties of the sensor array.

The capacitance signal was measured using an LCR meter (E4980AL, Keysight) at 1 kHz in a C_p mode. An LCR meter with a higher sampling frequency (TH2840B, Tonghui) was employed to determine the response time, because this machine has a fast sampling rate. The external force was applied using the force gauge (XLD-20E, Jingkong Mechanical Testing Co., Ltd.).

Articular pressure distribution test.

A Small Tail Han Sheep weighing 61 kg was selected as the animal model. After the immobilization of the sheep on a table, anesthesia was administered via intramuscular injection of xylazine, lasting for 3 h. After the knee joint is exposed, bone screws and sutures were used to fix the sensor array into the articular plane of the tibia. The animal experiments were performed in accordance with the protocol approved by the Institutional Review Board of the Southern University of Science and Technology, Shenzhen, China under protocol no. B202303-3.

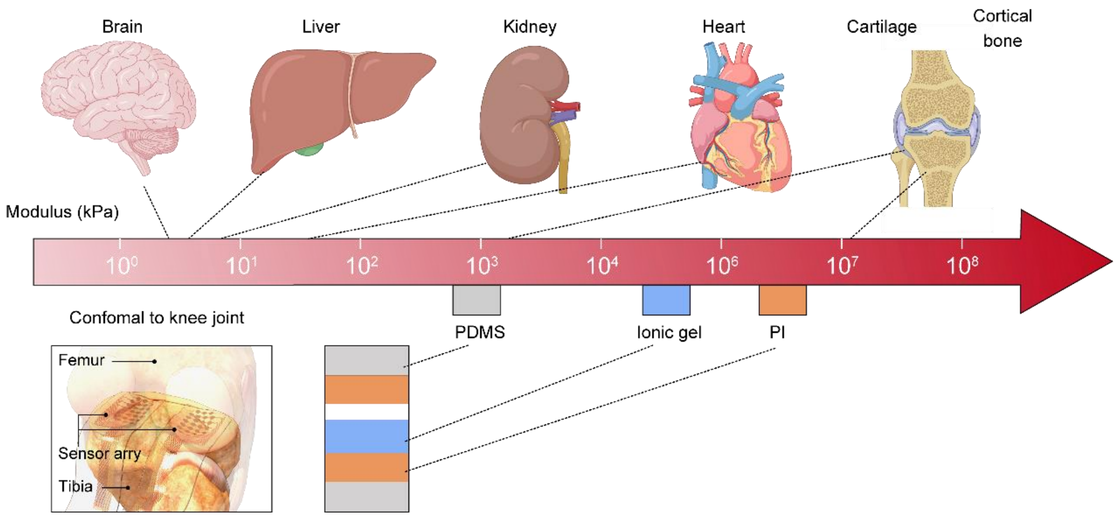

Figure S1. Comparison of the moduli of all sensor components and that of biological tissues. The sensing array pressure is a soft and thin layer, which can be conformally integrated on the cortical bone.

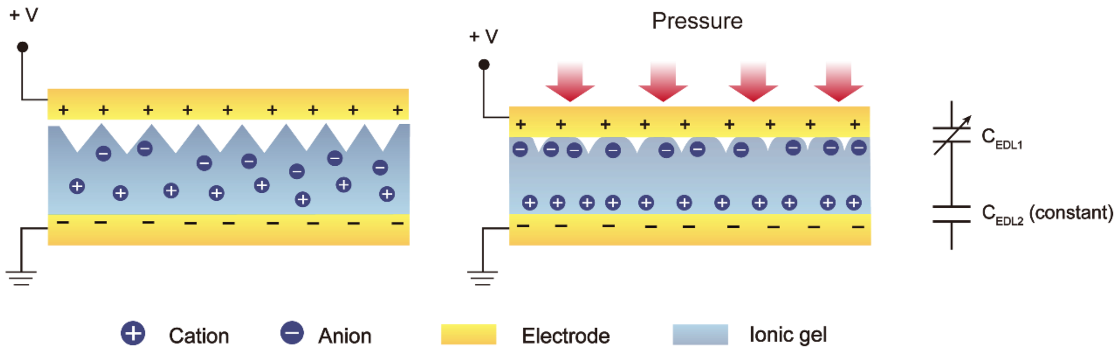

Figure S2. Sensing mechanism of the iontronic sensor. An electric double layer (EDL) will be built at the interface between the electrode and the ionogel. The contact area between the electrode and the microstructured ionogel varies as pressure changes, leading to the change in EDL capacitance (C_{EDL1}). The EDL capacitance (denoted as C_{EDL2}) for the flat interface does not change in varying pressures.

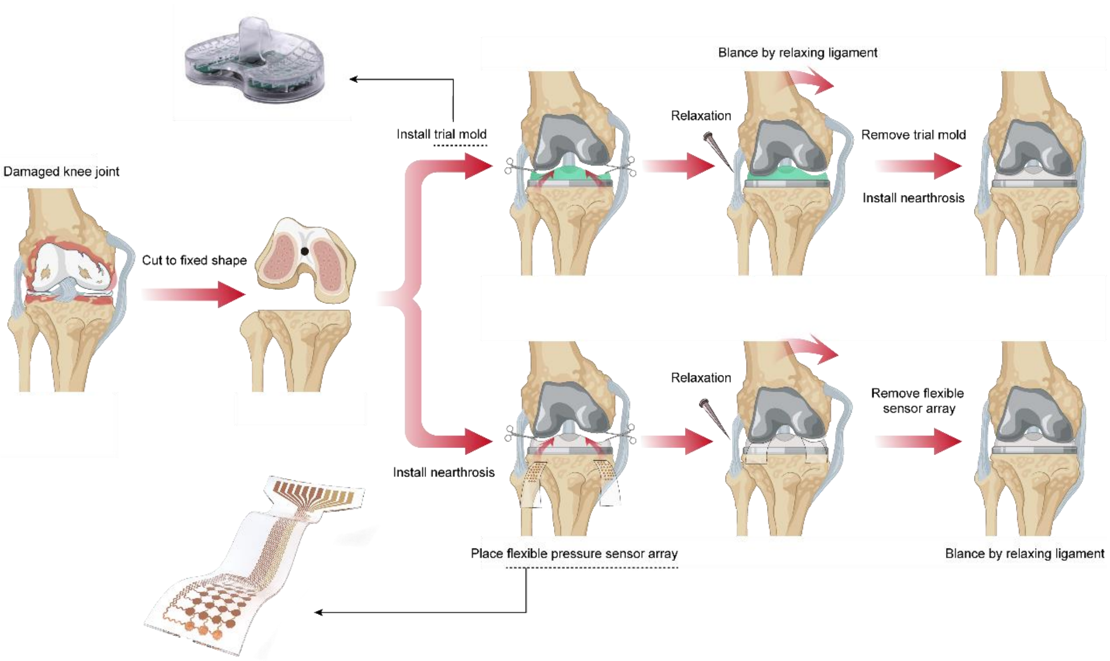

Figure S3. Comparison of the commercial trail mold and flexible pressure sensor array used to detect the intra-articular pressure. The up routine shows the surgical procedure of traditional intra-articular pressure detection during the total knee replacement, and the bottom routines shows the method of this study.

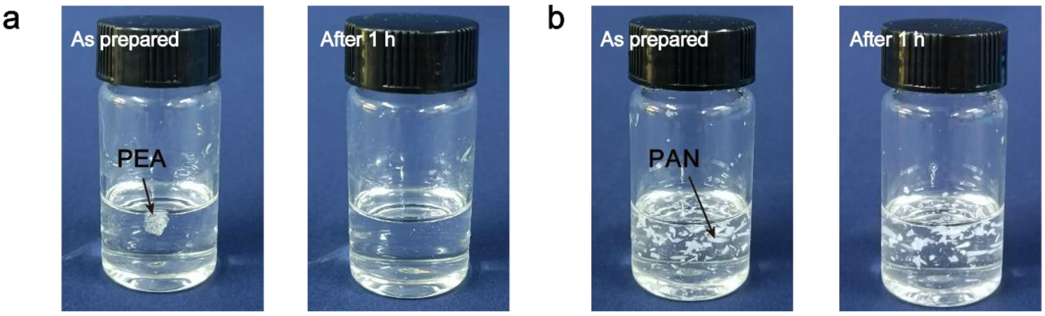

Figure S4. Differences in solubility of two polymers in ionic liquid [EMIM][TFSI]. (a) PEA can be easily dissolved in the ionic liquid. (b) PAN is almost insoluble in the ionic liquid.

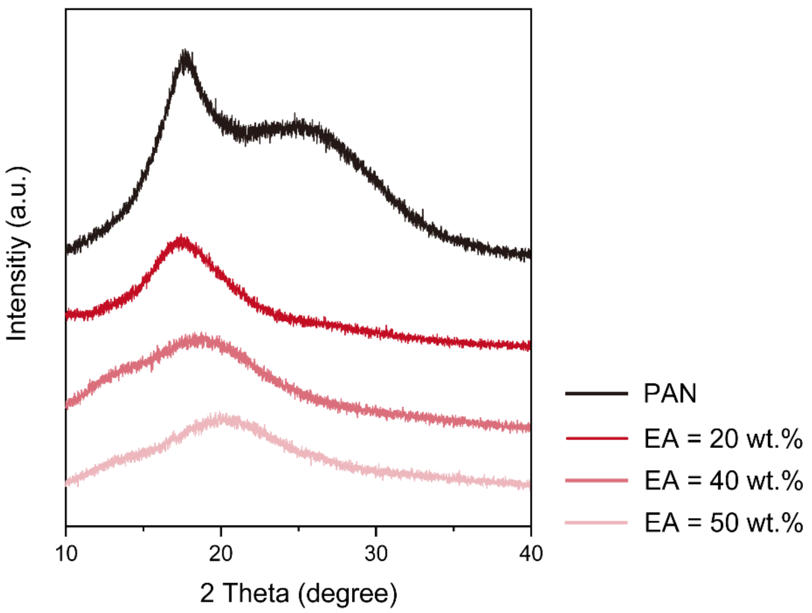

Figure S5. XRD results of the ionogels with different compositions. The crystalline degree enhanced with the increasing content of AN. Besides, the spectra demonstrate that the diffraction peak of the phase separation ionogels shifts left with increasing ionic liquid content because of the expansion of the lattice.

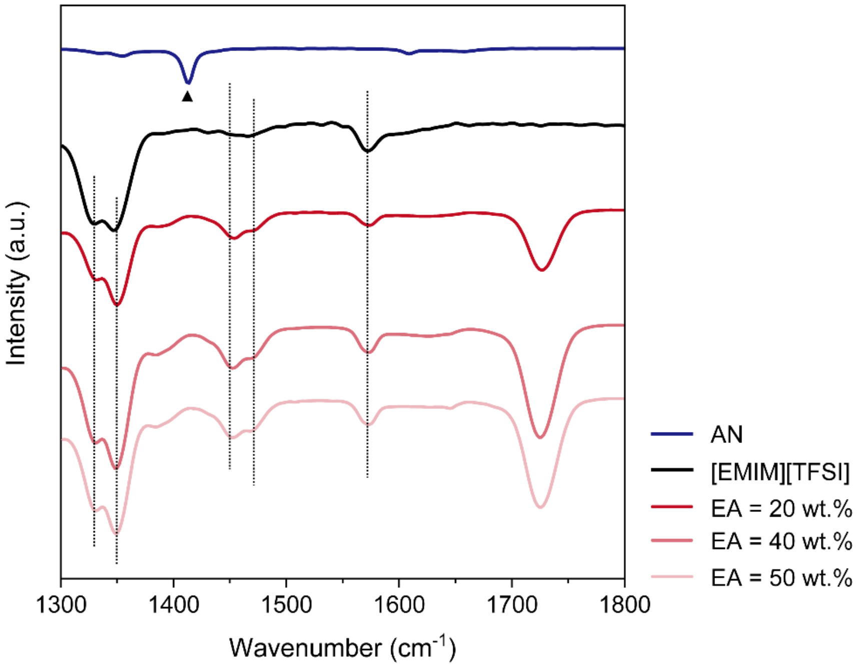

Figure S6. FTIR spectra of the ionogels with different compositions. The Peak at 1330 cm^{-1} represents the $\nu_{s,ip}$C-N, τCH_2 anf $\nu_{as,oop}SO_2$ from ionic liquid. The peak at 1350 cm^{-1} represents $\nu_{as,ip}$C-N, ωCH_2 adn $\nu_{as,ip}SO_2$ from ionic liquid. The peak at 1450 cm^{-1} represents $\delta_{sci}CH_2$ from imidazole ring in ionic liquid. The peak at 1471 cm^{-1} represents τCH_3 from imidazole ring in ionic liquid. The peak at 1570 cm^{-1} represented σCH_2 from imidazole ring in ionic liquid. The black triangle marked peak at 1411 cm^{-1} represents δCH_2 from AN. The subscripts “as” means “asymmetrical”, “s” means “symmetrical”, “ip” means “in-plane”, “oop” means “out-of-plane”, “ν” means “stretch”, “ω” means “wagging”, “δ” means “in-plane bending”, “τ” means “twist”, and “σ” means “scissor” [1,2].

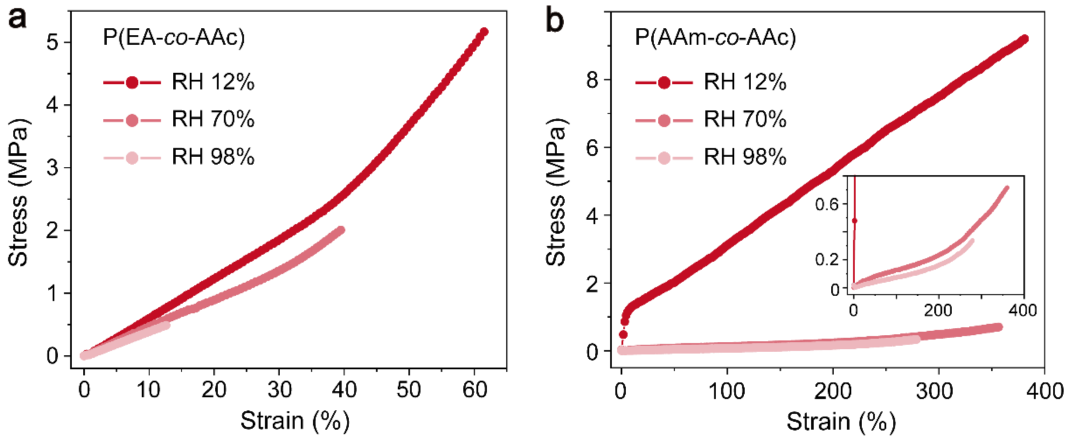

Figure S7. Mechanical properties of two control ionogels under different relative humidity levels. (a) Strain-stress curves of the poorly hydrophilic ionogel P(EA-co-AAc) at different relative humidity levels of RH 12%, RH 70%, and RH 98%. (b) Strain-stress curves of the highly hydrophilic ionogel P(AAm-co-AAc) at different relative humidity levels of RH 12%, RH 70%, and RH 98%.

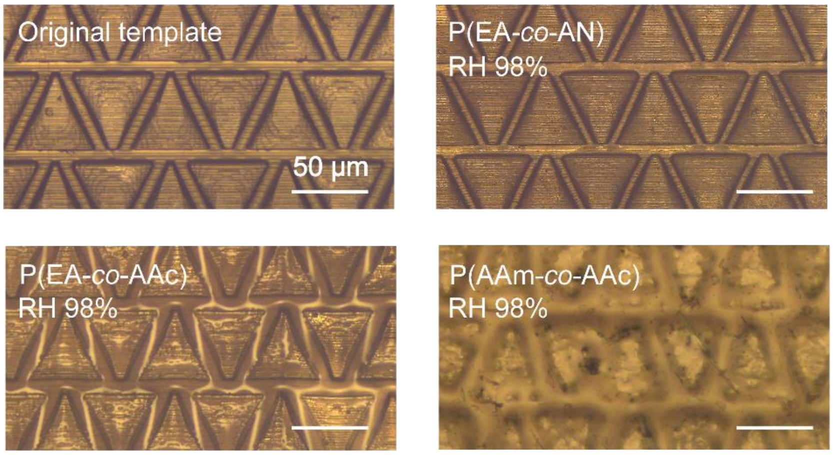

Figure S8. Morphological changes of the microstructures on the various ionogels under high humidity level of RH 98%. The morphology of the microstructures of the P(EA-*co*-AN) ionogel does not change, while that of the two control hydrophilic ionogels (P(EA-*co*-AAc) and P(AAm-*co*-AAc)) show the fusion of the microstructures in a high humidity environment of RH 98%.

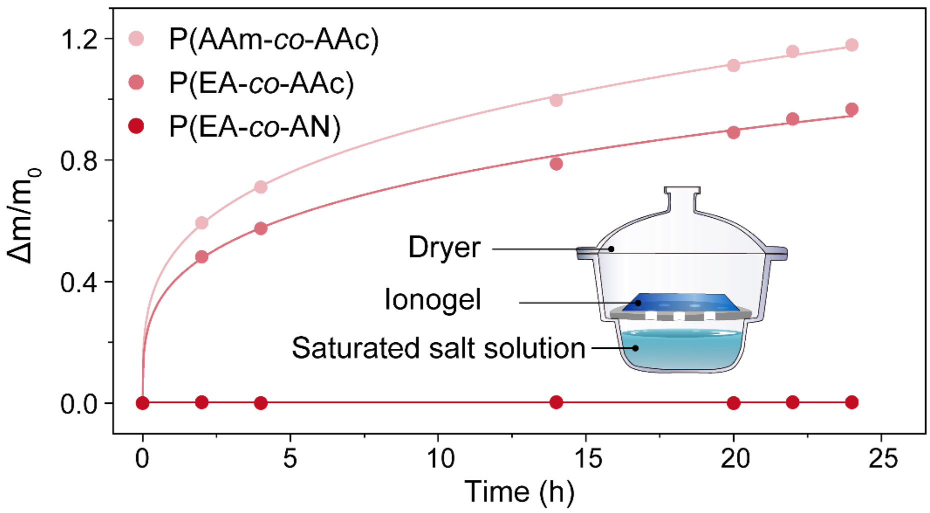

Figure S9. Mass changes of various ionogels under high humidity environment. The mass of the P(EA-*co*-AN) ionogel does not change with time, while that of P(AAm-co-AAc) and P(EA-co-AAc) have a large change over 24 h. The high humidity is generated using saturated salt solution in a sealed container.

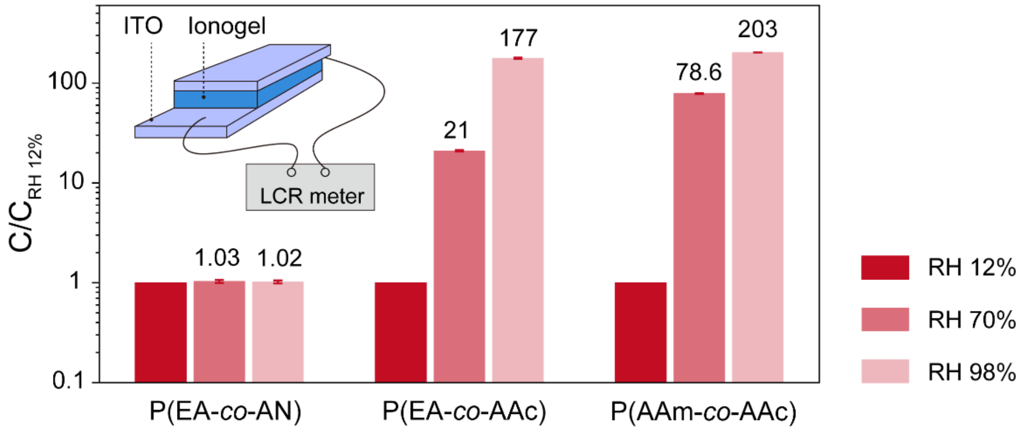

Figure S10. Relative capacitance in capacitors using the three ionogels under different relative humidity levels of RH 12%, RH 70%, and RH 98%. The P(EA-co-AN)-based capacitor has almost no signal drift, while that using the other ionogels have signal drift ratio of 1~2 orders of magnitude.

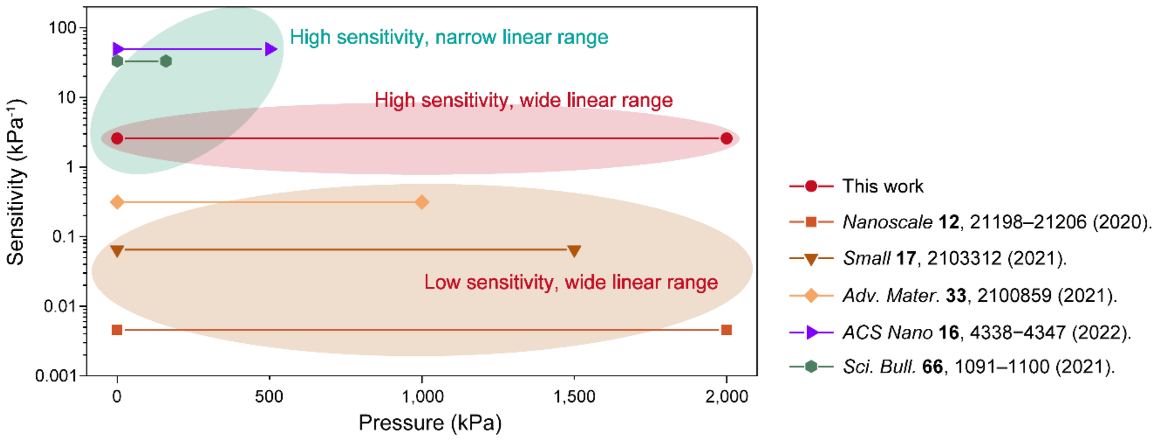

Figure S11. Comparison of sensitivity and linear working range of our iontronic pressure sensor and reported sensors in literature [3-7].

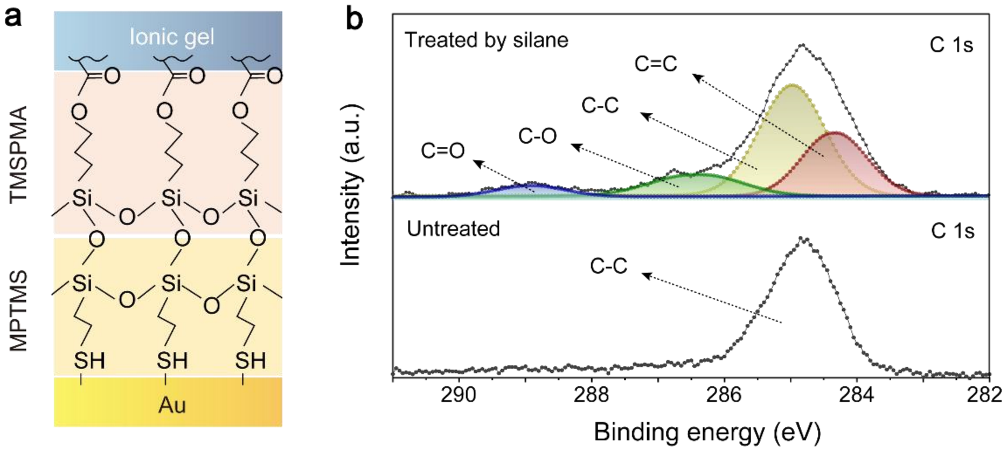

Figure S12. Surface modification of the interlayers of the sensor. (a) Schematic of the silane-based bridge bonding between the ionogel and the gold surface. (b) XPS spectra of the surface after modification by two types of silanes [8].

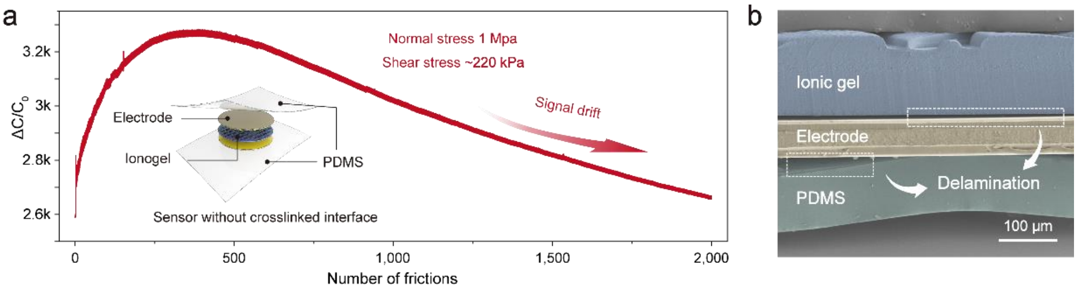

Figure S13. Stability in sensing signal and structure under rubbing cycles for the control sensor without crosslinked interfaces. (a) Response of sensor without interfacial bonding under repeated rubbing for 2,000 times at a normal pressure of 1 MPa and a shear stress of 220 kPa. (b) SEM images of the cross-section of the sensor after rubbing test, showing delamination of the interlayers.

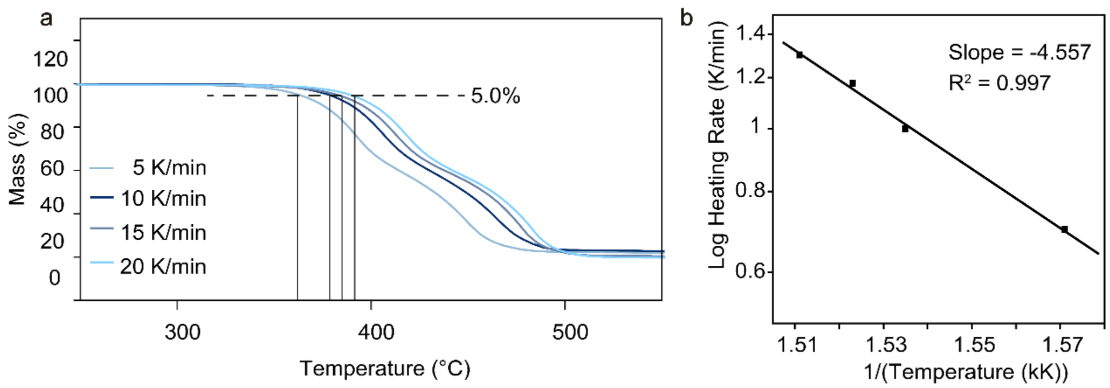

Figure S14. Thermogravimetric test of the P(EA-*co*-AN) ionogel. (a) Mass-temperature curves of the P(EA-co-AN) ionogel at different heating rates. **(b)** Fitted plot of the logarithm of the heating rate to the inverse of the temperature (kK). The data can be used to determine the activation energy of for degradation and acceleration factor in an aging test based on the protocol ASTM E1641-18.

The protocol is a standard test for decomposition kinetics by thermogravimetric analysis using the Ozawa, Flynn, and Wall's method [9], and determination of the acceleration factor for the P(EA-*co*-AN) ionogel is based on the Hallberg-Peck model [10]. The activation energy (E_{ad}) is computed from Eq. (1):

$$E_{ad} = -\left(\frac{k \cdot N_A}{b}\right) \cdot \Delta log[\beta]/\Delta\left(\frac{1}{T}\right) \quad (1),$$

where k and N_A represent Boltzmann's constant (8.6×10^{-5} eV K^{-1}) and Avogadro's constant (6.02×10^{23}), respectively, and b is the logarithm of the approximation derivative in K min^{-1}, β is the heating rate, and T represents the absolute temperature in Kelvin.

The mass losses of the P(EA-*co*-AN) ionogel at different heating rates (5, 10, 15, and 20 K min^{-1}) were measured. The mass loss curves at different heating rates are shown in **Fig. S14a**. The decomposition temperature of the P(EA-*co*-AN) ionogel is that when the mass drops by 5%. The Arrhenius plot of the decomposition temperatures and the heating rates are plotted in **Fig. S14b**, with the slope to be $\Delta log[\beta]/\Delta\left(\frac{1}{T}\right)$. By substituting this value in Eq. (1) with an initial b value of 0.457 (provided by ASTM E1641-18), an E_{ad} value of 0.8 eV was determined after quartic iterating.

The acceleration factor (AF) of the P(EA-*co*-AN) ionogel was computed based on the Hallberg-Peck mode, which considers the effect of both temperature and humidity (Eq. (2)).

$$AF = exp\left[\frac{E_a}{k} \cdot \left(\frac{1}{T_{normal}} - \frac{1}{T_{stress}}\right)\right] \cdot \left(\frac{RH_{stress}}{RH_{normal}}\right)^n \quad (2),$$

where E_{ad} is the activation energy determined to be 0.8 eV, n=3, RH_{stress} is the relative humidity of the accelerated level (RH_{stress}=98%), and RH_{normal} is the relative humidity at the normal condition (50%); T_{stress} is the temperature in Kelvin of the accelerated level (328 K), and T_{normal} is the temperature of the normal condition (298 K). AF was determined to be 131.

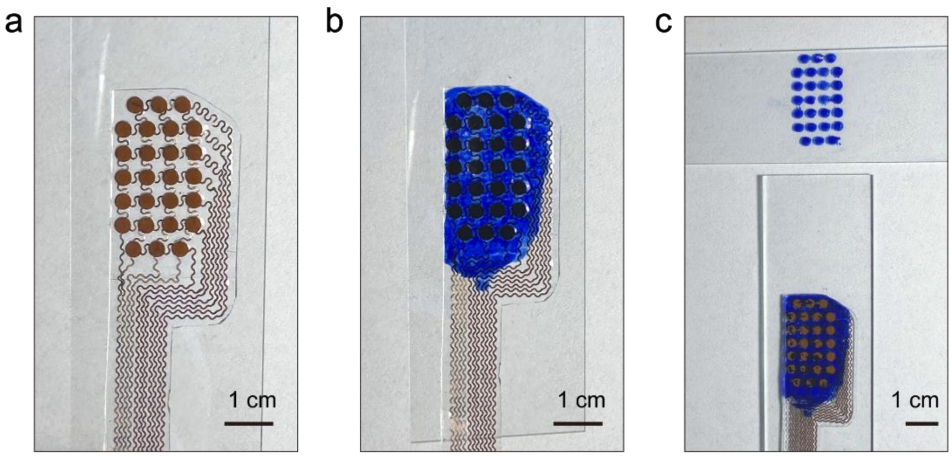

Figure S15. Photograph of contact area when applying a pressure. (a) Photograph of pre-tested sensor array. (b) Photograph of the device after staining. (c) Stained pattern on the glass after separation.

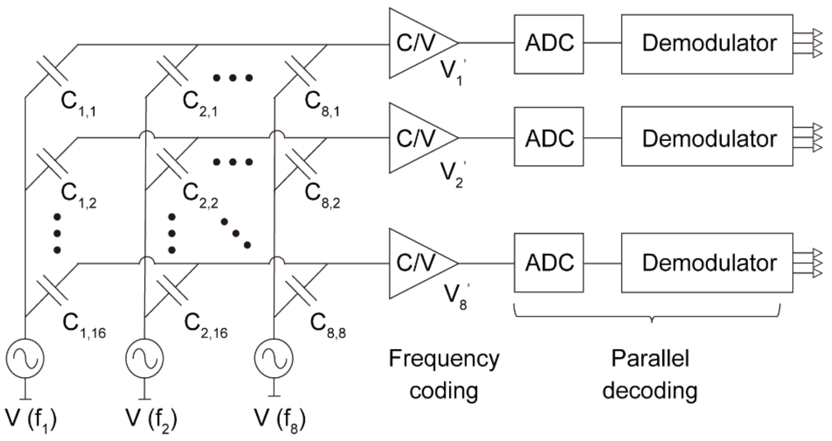

Figure S16. Schematic diagram of the acquisition circuit. Each row is read using an encoded frequency and the orthogonal frequency is propagated to the decoder using a capacitor-voltage (C/V) converter. Parallel decoding is realized through digital-to-analog converters (ADC) and a demodulator. $V(f_i)$ represents the input voltage with the orthogonal frequency f, while the V'_j is the voltage after frequency coding. $C_{i,j}$ is the capacitance value of the sensor. Subscripts i and j represent the row and column number of the sensors in the array.

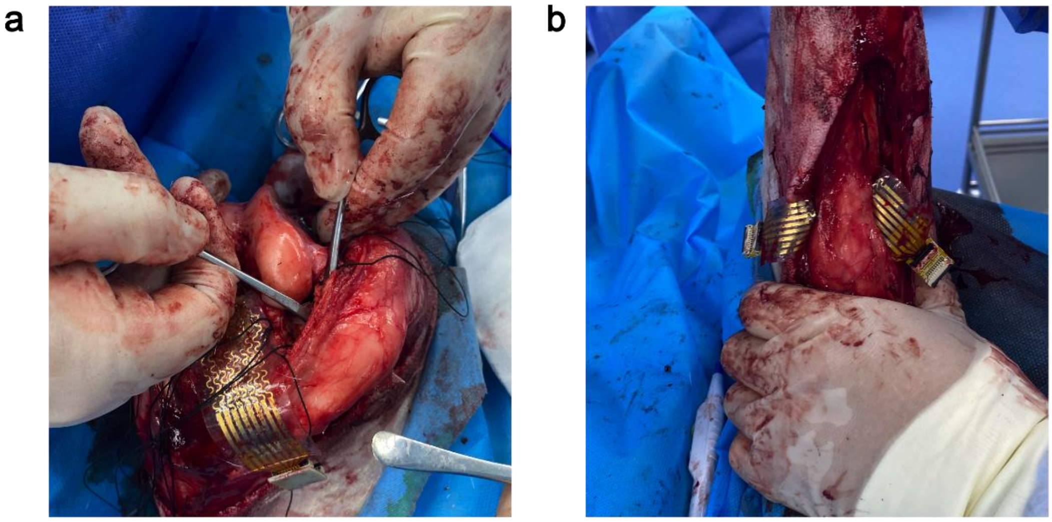

Figure S17. Implantation of the flexible sensor array in the knee joint of a sheep model. (a) The sensor arrays were fixated on the tibia surface of a sheep model using sutures and nails by a surgeon. (b) Two sensor arrays implanted in the knee joint.

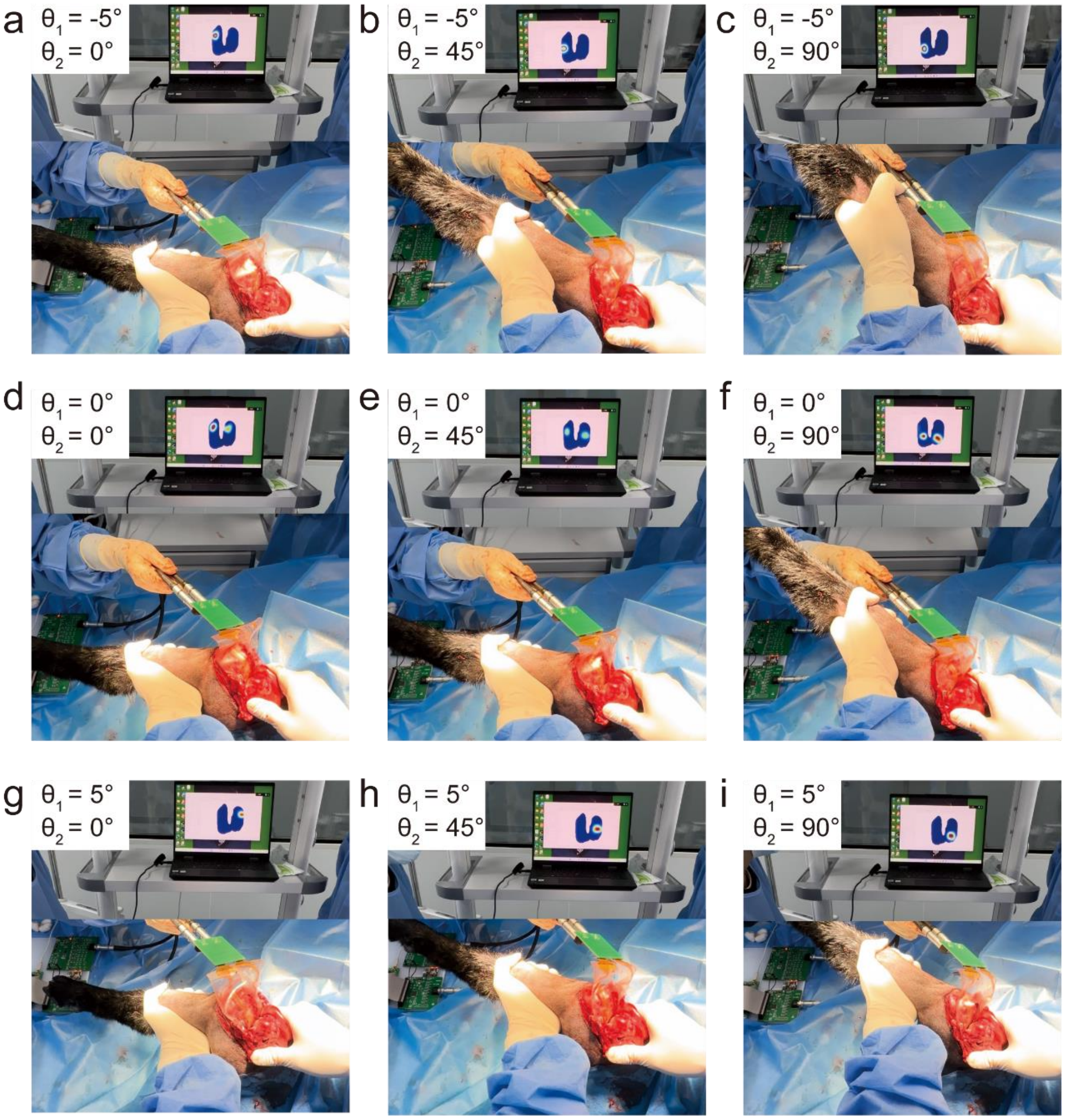

Figure S18. Photographs of nine combined states with θ_1 of –5 °, 0, and +5 °, and θ_2 of 0, 45 °, and 90 °. (a-c) States at θ_1=–5 °and θ_2=0, 45 °, and 90 °. (d-f) States at θ_1=0 °and θ_2=0, 45 °, and 90 °. (g-i) States at θ_1=5 °and θ_2=0, 45 °, and 90 °.

Supplementary References:

1. Arellano, I. H., Huang, J., & Pendleton, P. Computational insights into the molecular interaction and ion-pair structures of a novel zinc-functionalized ionic liquid, [Emim]$[Zn(TFSI)_3]$. *Spectrochim. Acta, Part A.* 2016; **153**: 6.
2. Clarke‐Hannaford, J., Breedon, M., Rüther, T., Johansson, P., & Spencer, M. J. Spectroscopic and computational study of boronium ionic liquids and electrolytes. *Chem. Eur. J.* 2021; **27**: 12826–12834.
3. Ji, B., et al. Bio‐inspired hybrid dielectric for capacitive and triboelectric tactile sensors with high sensitivity and ultrawide linearity range. *Adv. Mater.* 2021; **33**: 2100859.
4. Ji, B., et al. Gradient architecture‐enabled capacitive tactile sensor with high sensitivity and ultrabroad linearity range. *Small.* 2021; **17**: 2103312.
5. Wu, J., et al. Rational design of flexible capacitive sensors with highly linear response over a broad pressure sensing range. *Nanoscale.* 2020; **12**: 21198–21206.
6. Bai, N., et al. Graded interlocks for iontronic pressure sensors with high sensitivity and high linearity over a broad range. *ACS Nano.* 2020; **16**: 4338–434.
7. Lu, P., et al. Iontronic pressure sensor with high sensitivity and linear response over a wide pressure range based on soft micropillared electrodes. *Sci. Bull.* 2021; **66**: 1091-1100.
8. Qu, S., et al. High-performance n-type Ta 4 SiTe 4/polyvinylidene fluoride (PVDF)/graphdiyne organic–inorganic flexible thermoelectric composites. *Energy Environ. Sci.* 2021; **14**: 6586–659.
9. ASTM E1641–18. Standard test method for decomposition kinetics by thermogravimetry using the Ozawa/Flynn/Wall method. Standard, West Conshohocken 2018, PA.
10. Gao, Z., Yin, X., Zhang, B., Chen, M., & Li, B. A Wiener process–based remaining life prediction method for light-emitting diode driving power in rail vehicle carriage. *Adv. Mech. Eng.* 2019; **11**: 1687814019832215.
